# A Chinese drug-compatibility-based approach to purslane hydrogels for acute eczema therapy

**DOI:** 10.3389/fphar.2025.1504120

**Published:** 2025-02-05

**Authors:** Ling Wang, Yuzhong Zhang, Shenglin Geng, Lan Ma, Yiran Wang, Dongxu Han, Guojuan Fan, Weifen Zhang, Yanna Lv, Jinlong Ma

**Affiliations:** ^1^ School of Pharmacy, Shandong Second Medical University, Weifang, Shandong, China; ^2^ Dermatology, Weifang Hospital of Traditional Chinese Medicine, Shandong Second Medical University, Weifang, Shandong, China; ^3^ Collaborative Innovation Center for Target Drug Delivery System, Shandong Second Medical University, Weifang, Shandong, China; ^4^ Shandong Engineering Research Center for Smart Materials and Regenerative Medicine, Shandong Second Medical University, Weifang, Shandong, China

**Keywords:** purslane, eczema, hydrogels, tannins, drug compatibility

## Abstract

Purslane (*Portulaca oleracea L.*) with heat-clearing and detoxicating, anti-inflammatory and resolving swelling, relieving itching and astringing function, has remarkable efficacy for acute eczema. However, most of the clinical applications of purslane are freshly prepared decoction, not as easy to apply as cream, because the decoction is easy to breed bacteria and easy to oxidize. Here, based on the theory of Chinese medicines compatibility, we made a purslane-tannic acid hydrogel (PL-HATA) by simple methods under mild conditions to solve the drawbacks of easy oxidation and inconvenience of use of Purslane. The antimicrobial activity of PL-HATA hydrogel can exert an excellent antimicrobial effect, reducing the flora on the skin of acute eczema and further relieving the symptoms of acute eczema. At the same time, it creates a normal reactive oxygen species (ROS) microenvironment for acute eczema and promotes recovery from acute eczema. It also improves the symptoms of acute eczema by promoting cell proliferation and migration. Importantly, it resulted in improved skin lesion scores, scratching behavior, eosinophil infiltration, swelling and inflammation levels, immune homeostasis, and histopathological changes in rats with acute eczema. Besides, HATA hydrogel is not only suitable for Purslane’s decocted metabolites but also for Purslane’s freshly squeezed metabolites. This purslane application protocol solved the drawbacks of Purslane’s decoction, improved its storage stability and convenience of use, which is the key issue to further promote its clinical application.

## 1 Introduction

Acute eczema is a recurrent inflammatory skin disease affecting the superficial layers of the epidermis and dermis. It is primarily characterized by widespread, polymorphic, symmetrical erythema, papules, skin itching, dryness, inflammation, and other symptoms (Agner and Elsner, 2020). Eczema is a skin disease caused by various internal and external factors, with a complex pathogenesis. Clinically, antihistamines, calcium supplements, vitamins, antibiotics, and corticosteroids are primarily used for treatment (Weidinger and Novak, 2024). Long-term use of these non-specific medications can lead to adverse reactions such as skin wound infections and skin atrophy, and the long-term effectiveness is not significant. Traditional Chinese medicine has significant advantages in treating eczema, characterized by its safety, effectiveness, and minimal side effects. Traditional Chinese external therapies include external washing, wet compresses, dry compresses, paste methods, acupuncture, and others. However, these methods are complex to administer, inconvenient to use, and have relatively slow therapeutic effects. Purslane contains a large amount of polysaccharides, volatile oils, amino acids, and other effective components, which have anti-itching and astringent functions and are significantly effective for eczema treatment (Hon et al., 2011). Therefore, this study designed a purslane hydrogel, innovating the formulation of purslane and optimizing its components. This not only addresses the inherent drawback of purslane being easily oxidized but also resolves the adverse reactions and drug dependence caused by Western medicine treatments, as well as the inconvenience of using traditional Chinese medicine external treatments (Hwang and Lee, 2024; Li et al., 2024).

Purslane (*Portulaca oleracea L.*), a homology of medicine and food plant, has been listed as one of the most commonly used medicinal plants by the World Health Organization due to its good safety and few side effects (Xu et al., 2023). Purslane was first published in the Annotations to “Emperor Shen Nong’s Materia Medica” as a heat-clearing and detoxicating traditional Chinese medicine, which has the efficacy of dispersing the blood and resolving swelling, clearing the intestines and promoting diuresis, clearing away heat and reducing fire (Parham et al., 2020). This it can be used in the treatment of eczema and erysipelas and other conditions. In the Tang Dynasty purslane was used in the treatment of eczema, modern clinical application further proves that it can be effective in treating acute eczema. Modern pharmacology proves that purslane has the functions of antibacterial, relieving itching, anti-inflammatory, immune regulation, and repair of the skin stratum corneum barrier, which can be used to treat eczema (Al-Quwaie et al., 2023). However, most of the clinical applications of purslane involve the use of an external application of freshly prepared decoction, which is not as easy to apply as cream and is prone to spoilage and decay if not stored properly.

Purslane has many important active ingredients, such as flavonoids, alkaloids, terpenoids, organic acids, and polysaccharides (Iranshahy et al., 2017), which can be used to treat eczema. Among the flavonoids, the main ones are kaempferol, quercetin, apigenin, luteolin, and myricetin; alkaloids are mainly dopamine and norepinephrine (Yang et al., 2022); and terpenoids are ursolic acid and purslane monoterpenoids (Zhou et al., 2015). Most of the actives such as flavonoids, alkaloids, ursolic acid, and unsaturated fatty acids in purslane are susceptible to oxidization, which is most likely the essence reason for freshly prepared decoction of purslane for immediate use. Tannic acid (TA) are inexpensive plant-derived polyphenols, the active ingredients of the Chinese medicine gallnut, which can prevent oxidative stress and tissue damage and have a number of beneficial qualities, including non-toxicity, biocompatibility, antioxidant, antibacterial, and antigenic activities (Daré et al., 2020). In traditional Chinese medicine, gallnut is an astringent, which can reduce the exudation of acute eczema. According to the compatibility theory in traditional Chinese medicine, the compatibility of purslane and TA can play the roles of heat-clearing and detoxicating, astringing and resolving swelling, reducing local exudation. Importantly, the antioxidant function of TA can effectively prevent the oxidative inactivation of the active ingredients in purslane, and enhance the effectiveness of purslane in the treatment of eczema by balancing the ROS microenvironment. Therefore, inexpensive, plant-derived TA is the ideal compatibility drug for preparing purslane hydrogels, offering not only antioxidant properties but also the potential to enhance their therapeutic effects when combined with purslane (Lou et al., 2018).

Another difficulty encountered in the clinical application of purslane is that its application is decoction liquid, namely aqueous agent (Ning et al., 2023). Aqueous agent is not only inconvenient to use and poor adhesion, but also will affect its curative effect (Gallo et al., 2017). Hydrogels are considered ideal topical excipients because they have a highly moisturizing environment that mimics the extracellular matrix of the skin and protects wounds from bacteria and microorganisms (Chen et al., 2021; Gwak et al., 2021a; Qi et al., 2022; You et al., 2022; Guo et al., 2023; Zou et al., 2023). In addition, hydrogels generally have adhesion function, which can prolong the action time of drugs on lesion location (Guo et al., 2022). Hyaluronic acid (HA), the primary building block of the human extracellular matrix (Jeong et al., 2017; Wang et al., 2022), possesses anti-inflammatory and moisturizing properties, is biocompatible and biodegradable, and can shield skin from free radical damage (Hu et al., 2022; Tang et al., 2024). HA is widely used in biomedical applications such as cosmetics, dermal fillers, and tissue engineering, and is the ideal substrate for hydrogels (Zhai et al., 2020). Moreover, HA can be prepared into hydrogels with TA by hydrogen bonding and ionic bonding cross-linking (Zhu et al., 2018).

**Scheme 1:**
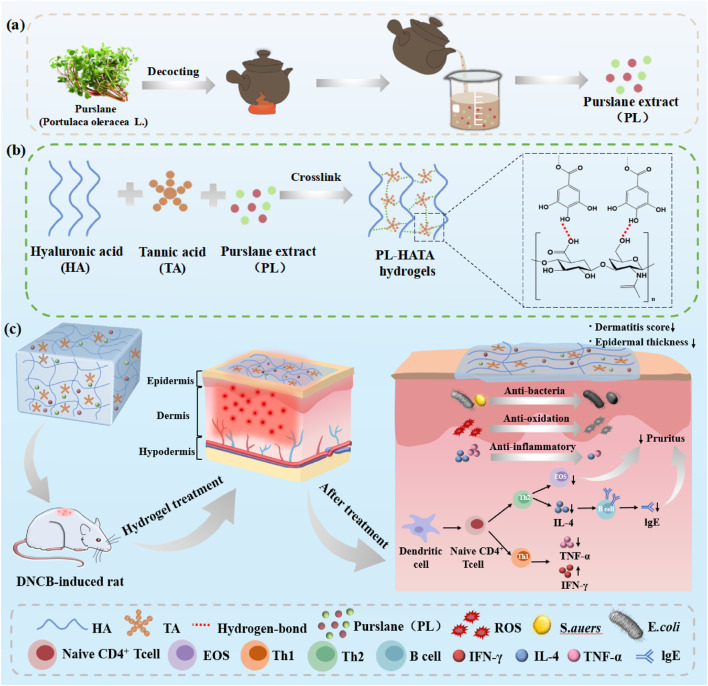
A review of purslane-tannic acid hydrogels and their applications. **(A)** Purslane decoction process; **(B)** Schematic diagram of the interactions between the components of purslane-tannic acid hydrogel; **(C)** Application of purslane-tannic acid hydrogel as a transdermal patch in a rat model of acute eczema.

Here, purslane-tannic acid hydrogel (PL-HATA) will be prepared by hydrogen bonding of TA and HA with a simple method in a short time under mild conditions for the treatment of 2,4-dinitrochlorobenzene (DNCB)-induced acute eczema in rats (Scheme 1). This hydrogel can address the issues of inconvenient use and poor adhesion associated with purslane lotion, and can also prolong the action time of the drug. The antioxidant and antibacterial effects of TA can achieve long-term storage stability of Purslane hydrogels and prevent local infection in eczema (Heydarirad et al., 2024). PL-HATA can also create a suitable ROS microenvironment for acute eczema by regulating the oxidative stress balance. This study will provide a research idea for the clinical application of purslane and a practical solution for its use in the treatment of acute eczema, which has important theoretical and practical significance for promoting the development of Chinese medicine.

## 2 Experimental

### 2.1 Materials

HA (Mw = 40–80 kDA), TA (Mw: 1701 Da), 2-(N-morpholino)ethanesulfonicacid (MES), 2.4-dinitrotoluene were purchased from Shanghai Maclean’s Biochemical Science and Technology Co., Ltd., IFN-γ and IgE ELISA kits were purchased from Quanzhou Ruixin Bio-technology Co. Ltd.3-(4,5-dimethyl-2thiazolyl)-2,5-diphenyl-2-h -tetrazolium bromide (MTT) and Calcein-AM/PI double staining kit were purchased from Shanghai Yisheng Biotechnology. L929 cells were obtained from American Type Culture Collection (ATCC, Manassas, VA, United States). Standard strains of *Escherichia coli* (*E. coli*, cat. no. ATCC8739) and *Staphylococcus aureus* (*S. aureus*, cat. no. ATCC 7755) were purchased from Beijing AnxinKang Science and Technology Co. and were stored at −20°C until use. The remaining reagents were analytical grade reagents (e.g. Luria Broth, Agar, etc.) and were used without further purification.

### 2.2 Methods

#### 2.2.1 Drug preparation

About 100 g of fresh purslane was weighed, cut and ground in the juicer, adding distilled water for juicing to obtain a crude liquid with a low concentration equivalent to about 0.3 g/mL of crude drug content. The crude fluid was then filtered through five layers of sterile gauze to obtain the fresh purslane medicinal liquid (PL-f). Another 100 g of fresh purslane was dried at 70°C–75°C to obtain about 10 g of traditional Chinese medicine purslane, which was accordingly decocted with distilled water for 10–15 min to obtain the same concentration of purslane decoction (PL). PL-*f* and PL were all freshly prepared, and left them at room temperature for 7 days to obtain 7d+PL and 7d+PL-*f*.

#### 2.2.2 Preparation of HATA hydrogel

The 1.95 g of MES powder was first dissolved in 100 mL of deionized water, ultrasonicated, and oscillated until all dissolved. The pH value of the solution was measured using a pH meter (pH 2.5). Then, 1 mol/L NaOH solution was slowly added to adjust the solution pH to 5.5, the volume was fixed, and the MES buffer (0.1 mol/L) was obtained. HA solution was prepared by dissolving HA in MES buffer solution (pH 5.5). The tannic acid was dissolved in the aqueous solution, and then the HA solution was mixed with the solution with different TA content (the molar ratio of HA:TA was 1:1, 1:2, 1:3), and 1 mL of glycerin was added. Finally, the mixed solution was placed in a centrifuge tube and centrifugated at 37°C at 10,000 rpm/min for 10 min to remove bubbles, and HATA hydrogel was prepared.

#### 2.2.3 Preparation of purslane-tannic acid hydrogel

According to the rheological properties of hydrogel in the following experiments, HATA hydrogel with better mechanical properties was selected, and 10 mL of purslane solution (PL-*f*, PL, 7d+PL, 7d+PL-*f*) was added according to the same steps as those for the preparation of HATA hydrogel above, thus getting PL-HATA, PL-*f*-HATA, PL-HATA and 7d+PL-*f*-HATA.

### 2.3 Characterization of HA-TA

The cross-sectional morphology of the hydrogels was analyzed using scanning electron microscopy (SEM) (Zeiss Merlin Compact, Germany) operating at an accelerating voltage of 5.0 kV. Before observation, the hydrogels were freeze-dried at −80°C for 3 days, and fractured using a blade to obtain dried hydrogel sheet (both length and width less than 1 cm), and coated with ∼30 nm coating layer use Au sputter (Gwak et al., 2021b; Liu et al., 2022). Pore size was measured using a software (ImageJ, National Institute of Health, United States). Changes in the chemical structure of HATA hydrogels were analyzed using attenuated total reflectance infrared spectroscopy (Thermo Scientific Nicolet iS30, United States). All hydrogels’ spectra were captured within the 500–4,000 cm^−1^ wavelength range (Raina et al., 2023).

In addition, the rheological properties of HATA hydrogels were evaluated using a rheometer (Haake Mars40, Germany). The measurement was carried out in strain:1%, frequency:0.1–10 rad/s for oscillatory frequency scanning. The viscoelastic behavior of HA/TA hydrogels was investigated based on storage modulus (G′) and loss modulus (G″) values. The ratio of elastic to viscous behavior was used to express the viscoelastic behavior, which was computed as G′/G″ (Gwak et al., 2021b). In order to allow sufficient physical cross-linking of HA and TA, the rheological properties were measured after the made hydrogels were stabilized for 1 h.

### 2.4 Storage stability experiment

The gelling agent product should maintain a uniform and fine gel, i.e., no delamination and caking phenomenon under centrifugation, low temperature, and high temperature. In order to test the stability of hydrogel, its stability was determined from these four angles.

(1) Centrifugation: 10.0 g of the hydrogels were taken into a centrifuge tube and centrifuged for 10 min at a speed of 3,000 r/min to see if there was any delamination.
(2) Low temperature: 10.0 g of the hydrogels were taken into a glass screw bottle and placed in a refrigerator at −20°C for 6 h with the lid closed to observe whether the phenomenon of delamination occurs.
(3) High temperature: 10.0 g of the hydrogels were taken and placed into a glass screw bottle, with the constant temperature oven pre-set to 55°C. The hydrogels were then covered with a lid and placed in the oven for 6 h to observe if delamination occurred.
(4) Bacterial experiment: PL, PL-*f*, 7d+PL, 7d+PL-*f*, HATA, PL-HATA, PL-*f*-HATA, 7d+PL-HATA, and 7d+PL-*f*-HATA (70 μL) were evenly dripped onto LB solid medium, and the samples were spread evenly with a sterile applicator stick until dry, PBS was used as a control. Then the plate was inverted in a constant temperature incubator for 24 h at 37°C overnight. Finally, the colony formation on the plate was observed to examine the stability of purslane storage.

### 2.5 Determination of cell viability

To detect the cytotoxic effect of purslane hydrogels on HaCaT cell viability, MTT assay was used to evaluate the effect of hydrogel on cell viability. Hacat cells were inoculated into 96-well plates at a density of 1 × 10^4^ cells/well. The cells were cultured in DMEM (37%) medium containing 15% FBS, 1% penicillin and streptomycin, and 1% sodium pyruvate and incubated in an incubator (5% CO_2_, 37°C) for 24 h, then the cells were treated with the different experimental groups for 24 h. The DMEM-treated cells were used as a negative control. 10 μL of MTT (5 mg/mL) reagent was added to each well and then incubated in the incubator for 3 h. Cell viability was evaluated by measuring optical density (OD) at 490 nm using a microplate reader (n = 3).

### 2.6 Live/dead cell staining assay

To better evaluate the biocompatibility of the cells, HaCaT cells were inoculated in 24-well plates (1.3 × 10^5^ cells/well) for live/dead staining assay. The HaCat cells were incubated in hydrogels for 24 h, and 48 h, in which DMEM-treated cells were used as the control, and HaCaT cells were stained with flavin acetoxymethyl ester calcium (AM)/propidium iodide (PI), and the cells were incubated for 15 min at room temperature under the condition of avoiding the light. The 24-well plates were imaged using an inverted fluorescence microscope (Leica DMI4000B, Germany), and cell activity and morphology were observed (n = 3).

### 2.7 Cell migration assay

HaCaT cells at the logarithmic growth stage were inoculated with 3 × 10^5^ cells in 6-well plates and incubated at 37°C for 24 h. The cell fragments were evenly marked in the orifice plate with a 10 μL spearhead and washed with sterile PBS, and the hydrogel solutions was placed into the wells. The growth and migration of cells around the scratch were observed at set times (24 h and 48 h) (n = 3).

### 2.8 DPPH free radical scavenging assay

Based on the existing literature, DPPH (1, 1-diphenyl-2-picrylhydrazyl) radical scavenging assay was used to evaluate the antioxidant activity of the hydrogels (Zhang et al., 2020; Xu et al., 2021). A 0.1 mM solution of DPPH was prepared by dissolving DPPH in methanol, the DPPH solution in methanol (1.6 mL) was mixed with methanol (0.4 mL) as a control. The DPPH solution (1.6 mL) was mixed with PL, PL-*f*, 7d+PL, 7d+PL-*f*, HATA, PL-HATA, PL-*f*-HATA, 7d+PL-HATA, and 7d+PL-*f*-HATA (0.4 mL) as experimental groups. The absorbance of the different groups was measured at 60 min. The absorbance was determined using a UV-Vis spectrophotometer (Evolution 300, Thermo, United States) in the 400 ∼ 800 nm wavelength range. After an additional 30 min incubation in a dark box at room temperature, the change in absorbance of the pre-existing hydrogels was monitored at 517 nm using a Multiskan FC (Thermo, America) microplate analyzer.

Each group was repeated three times, and the DPPH radical scavenging effect was calculated as shown in Equation 1:

$$\text{Scavenging activity}\left(\%\right)=\frac{A_{1}-A_{2}}{A_{1}}\times 100$$ (1)

where A_1_ is the absorbance of DPPH and A_2_ is the absorbance of DPPH radicals in the presence of the sample.

### 2.9 *In vitro* evaluation of ROS levels

In this study, the ability of purslane hydrogel to modulate ROS levels was investigated based on the model of H_2_O_2_-induced oxidative damage in HaCaT cells. Cells were inoculated into 24-well plates (1.3 × 10^5^ cells/well) at the logarithmic growth phase and cultured in the incubator at 37°C for 24 h. Then, the cells were treated with the hydrogel solutions for 6 h, and then H_2_O_2_ (15 μL) was added to act for 0.5, 1, and 2 h. The ROS assay was performed strictly according to the instructions, with the addition of 500 μL of diluted DCFH-DA per well (DCFH-DA: DMEM = 1:1,000 serum-free medium diluted to 10 mmol/L), incubated at 37°C for 30 min. Then washed gently with PBS for 1–2 times to remove the excess cellular DCFH-DA. Finally, the ROS content in each group was observed under inverted fluorescence microscope (n = 3).

### 2.10 Hemolysis experiment

The purslane-tannic acid hydrogel was incubated with rat erythrocytes to evaluate its hemolytic activity. Red blood cells (RBCs) were isolated from 2 mL of blood from healthy SD rats, centrifuged for 15 min at 2000 rpm, and then washed three times in sterile PBS washes. About 300 μL of RBCs were dispersed in 1.1 mL of PBS to obtain RBC suspension. The RBC suspension was added to each hydrogel solution, incubated at 37°C for 3 h, centrifuged at 11,000 rpm for 15 min, and the supernatant was collected. 100 μL of supernatant was taken from each group and transferred to a 96-well plate, and its absorbance at 540 nm was detected by an enzyme marker Multiskan FC (Thermo, America). The PBS and deionized water-treated groups were used as negative and positive controls.

Three replicates were used in each group, and the percentage of hemolysis per well was calculated as shown in Equation 2:

$$\text{Hemolysis }\%=\left(\text{Absample}-{\text{Ab}}_{\text{PBS}}\right)/\left({\text{Ab}}_{H2_{O}}-{\text{Ab}}_{\text{PBS}}\right)/100$$ (2)

### 2.11 *In vitro* evaluation of antimicrobial activity

Two bacteria, *E. coli* (*E. coli*, ATCC 8739) and *S. aureus* (*S. aureus*, ATCC 7755), were used as model bacteria for evaluatet. The antimicrobial properties of purslane-tannic acid hydrogel were evaluated. LB agar (Luria-Bertani agar) and LB broth (Luria-Bertani broth) were used as a source of bacterial nutrients (n = 3).

#### 2.11.1 Plate counting experiments

200 μL of bacterial suspension (10^8^ CFU/mL) was mixed with various solutions including PL, PL-*f*, 7d+PL, 7d+PL-*f*, HATA, PL-HATA, PL-*f*-HATA, 7d+PL-HATA, 7d+PL-*f*-HATA and PBS (900 μL), and PBS was used as a control. The mixtures were co-cultured at 37°C on a shaker for 12 h (100 rpm/min). Subsequently, the mixtures were diluted 10^6^ times using sterile PBS, and 60 μL was uniformly coated on a petri dish and cultured in an incubator at 37°C for 18–24 h. Ultimately, the petri dishes’ colony-forming units (CFU) were tallied (n = 3).

#### 2.11.2 Inhibition curve

In order to determine the specific duration of action of the purslane hydrogels, bacterial growth was detected in real-time using a bacterial inhibition curve. The bacterial solution (10^8^ CFU/mL, 10 μL) was added to 96-well plates with PL, PL-*f*, 7d+PL, 7d+PL-*f*, HATA, PL-HATA, PL-*f*-HATA, 7d+PL-HATA, 7d+PL-*f*-HATA and PBS (90 μL), where PBS was used as a control, and incubated at 37°C. OD values were measured at 600 nm every 2 h (0, 2, 4, 6, 8, 10, 12 h) using a Multiskan FC (Thermo, America) microplate analyzer (n = 3).

#### 2.11.3 Live and dead staining assay

In order to further verify the bactericidal effect of purslane hydrogels, “live/dead” bacteria were stained with a mixture of DMAO and EthD III dyes and analyzed by fluorescence detection (Pan et al., 2022; Zhu S. et al., 2023). The bacterial suspension (10^8^ CFU/mL, 1.6 mL) was mixed with PL, PL-*f*, 7d+PL, 7d+PL-*f*, HATA, PL-HATA, PL-*f*-HATA, 7d+PL-HATA, 7d+PL-*f*-HATA and PBS (0.4 mL), the sterile PBS was used as the control, and the incubation was carried out at 37°C for 12 h. 100 μL of bacterial solution from each group was transferred to a 96-well plate, 1 μL of mixed dye was added to each well, and incubated for 15 min at 37°C, protected from light, and the bacterial survival was detected under a fluorescence microscope (Leica DMI4000B, Germany). The fluorescence intensity was quantified by ImageJ software.

### 2.12 Study on the effectiveness of purslane-tannic acid hydrogel in the treatment of acute eczema

#### 2.12.1 DNCB-induced preparation of acute eczema model in rats

In order to further evaluate the therapeutic effect of purslane hydrogels on the effect of acute eczema, a DNCB-induced acute eczema model was established in 40 SD rats (males, 180–220 g, SPF grade), which were randomly divided into 10 groups. All animal experiments were approved by the Institutional Review Board of Shandong Second Medical University (2023SDL277). First, the abdominal hair of rats was removed, covering an area of about 2 cm × 3 cm, and the left and right sides of the back were removed, covering an area of about 2 cm × 2 cm. 5% DNCB 30 μL was applied to the abdominal skin for the first time sensitization. On the third day of the modeling, the shaved area of the right back was coated with 0.2% DNCB 50 μL for the second antigenic stimulation, and the symmetrically shaved area of the left-back was left without pharmacological sensitization as the control site. Afterward, the rats were stimulated every 3 days for 3 consecutive times. On the ninth day, it was observed that the right back of the rats was more swollen, erythematous, oozing, and scratching than the left back, and the increase of eosinophils and IgE in the serum confirmed the success of the model. Starting from the 10th day, each of the 10 groups was given 1 mL of medication (including saline, PL, PL-*f*, 7d+PL,7d+PL-*f*, HATA, PL-HATA, PL-*f*-HATA, 7d+PL-HATA, 7d+PL-*f*-HATA) twice a day, once in the morning and once in the afternoon, for 9 consecutive days, and the treatment group was the control group (Achdout et al., 2017).

#### 2.12.2 Quantitative analysis of dermatitis score and scratching behavior of rats

Beginning on the day of completion (day 10), erythema, papules, epidermal exfoliation, and scaling lesions were observed visually as dermatitis damage. Eczema was scored according to the severity of the symptoms: No symptoms, mild, moderate and severe symptoms were scored as 0, 1, 2 and 3 points respectively, and the sum of each symptom score was the total dermatitis score, which was ranged from 0 to 12, and the higher the score, the more severe the eczema (Mernelius et al., 2016; Klose et al., 2023). To quantify the symptoms of itching, the frequency of rats scratching the treatment site with their hind limbs was measured and recorded on 3 and 9 d of treatment and observed for 30 min.

#### 2.12.3 Analysis of skin swelling and skin roughness

A circular perforator (6 mm in diameter) was used to perforate the healthy skin on the left side and the skin of the modeling area to obtain the same area of skin, which was weighed with an analytical balance (unit of measurement mg).The skin Swelling and swelling ratio as shown in Equation 3:

$$\text{Swelling ratio }\left(\%\right)=\left(\text{inflammatory skin weight}-\text{healthy skin weight}\right)/\text{healthy skin weight}\times 100\%$$ (3)

In order to understand the skin roughness on the eczema surface, the rat skin in each group was quantified using a multifunctional skin tester at 3 and 9 d.

#### 2.12.4 Levels of serum eosinophils

The skin tissues fixed, dehydrated, embedded, and prepared for paraffin sections were dyed and sealed, and the dermis region was then selected for 400-fold imaging using an Eclipse Ci-L photographic microscope (Nikon, Japan). When capturing images, ensure that the tissue fills the entire field of view and that the background light is consistent across each photo. After imaging was completed, using Image-Pro Plus 6.0 analysis software, the number of eosinophils in 3 fields of view of each section was counted separately using millimeters as the standard unit, and the field of view area was measured.

#### 2.12.5 TNF-α, IL-4 content

Immunohistochemistry was used to determine the expression of TNF-α and IL-4 in skin tissues. The above-fixed, dehydrated, and embedded skin tissues were sectioned to create paraffin sections. TNF-α and IL-4 in these sections were stained using the IHC SP method (Kirsten et al., 2017; Wang et al., 2024). 4 primary antibodies (clone number: BA0980, rabbit anti-mouse IL-4 polyclonal antibody, purchased from Wuhan Doctor Bioengineering Co.) immunohistochemically stained sections of TNF-α and IL-4 were observed under the microscope. The distribution of the positive substances was determined as light yellow, yellow or brownish yellow filamentous or granular substances located in epidermal echinodermal cells, sweat gland cells, hair follicle cells, and other skin appendages, as well as the mesenchymal tissues within the dermis (Dávila-Martínez et al., 2020; Ekici et al., 2022). Finally, the integrated optical density (IOD) of TNF-α and IL-4 positive substances was measured with a pathology image analysis system to indicate the expression levels of TNF-α and IL-4.

#### 2.12.6 Expression levels of serum IFN-γ and IgE

Blood of anesthetized rats was taken from the eyeballs, left for 30 min, and centrifuged at 3,000 r/min for 10 min, supernatants were taken, and the expression levels of IFN-γ and IgE in serum of the 3 groups were detected by ELISA.

#### 2.12.7 Histological observation of rat back skin

The skin and organ samples were collected at the set time points (3 and 9 d), fixed in 4% paraformaldehyde, embedded in paraffin, and sectioned at a thickness of 5 μm. After hematoxylin and eosin staining (H&E) and Masson trichrome staining (MT) were used, the morphological changes of skin tissues were observed under the light microscope, so as to determine the degree of inflammatory activity of the tissues.

#### 2.12.8 Statistical analysis

Each experiment was repeated independently at least three times. All data were expressed as mean ± SD. Data were analyzed using GraphPad Prism. One-way analysis of variance (ANOVA) was used to assess the treatment effect. p < 0.05 was considered a statistically significant difference (*p < 0.05, **p < 0.01, ***p < 0.001).

## 3 Results and discussion

### 3.1 Characterization

**FIGURE 1:**
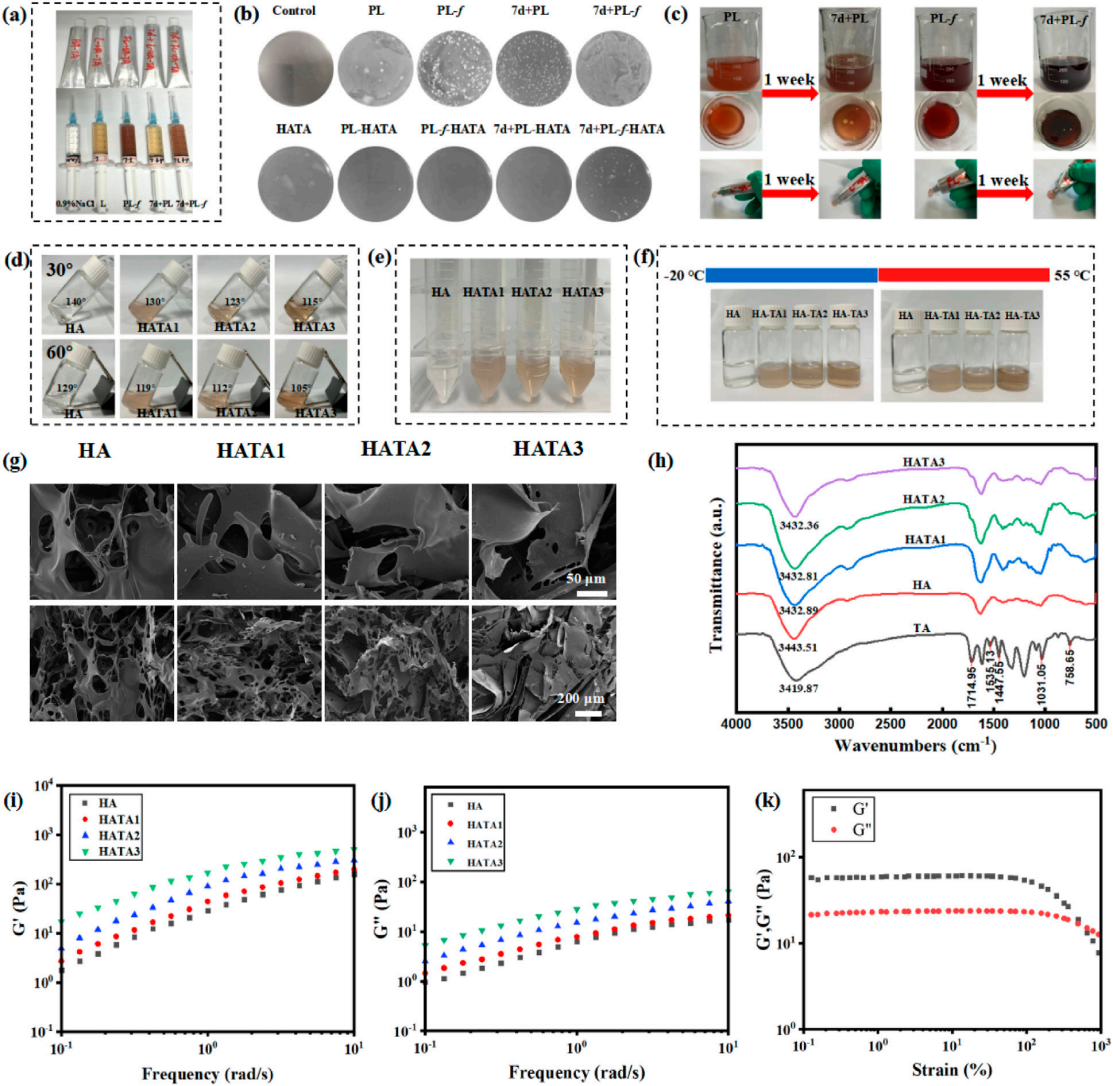
**(A)** Pictures of hydrogel application; **(B)** The stability of Purslane hydrogels was investigated by stored bacterial experiments. **(C)** Purslane solution placed for a week pictures. **(D)** Photographs of hydrogels at different tilt angles HATAs (molar ratios of 1:0, 1:2, 1:3, and 1:4). **(E)** Pictures of hydrogels subjected to centrifugation. **(F)** Photographs of hydrogels subjected to cold and heat resistance tests. **(G)** SEM images of HA, HATA1, HATA2, and HATA3. **(H)** FT-IR spectra of HATAs hydrogels; **(I)** Strain amplitude scanning test, **(J, K)** frequency scanning analysis of HATAs hydrogels.

Purslane hydrogel, with its good mobility, can be easily installed into the hose and is user-friendly when applied to the lesion site (Figure 1A). The results of stored bacterial experiments (Figure 1B) show that there is a significant difference between 7d+PL, 7d+PL-*f* and PL, PL-*f*. As can be seen from the plate, 7d+PL, 7d+PL-*f* easy to breed bacteria. By observing the state of the solution in the beaker (Figure 1C), it was found that 7d+PL and 7d+PL-*f* were prone to bacterial contamination when stored for too long. Therefore, direct use may cause potential impact on the skin and most of the clinical applications of purslane are freshly prepared decoction. When the purslane was made into a gel dosage form, and after a week of placement, the appearance did not change significantly and there was no bacterial contamination. It indicates that the gel dosage form could be stored for a long time, and this dosage form solved the disadvantages of the storage stability and inconvenience of purslane. The formation condition of HATA hydrogel could be observed by placing it at different tilt angles (Figure 1D). It can be seen from the figure that HA is in solution state. With the increase of TA content, the tilt angle of the HATA hydrogel becomes smaller, and its viscosity is enhanced. Particularly, HATA3 can form hydrogels with stable adhesion. The HATA hydrogels were subjected to relevant quality-examination, and the results showed that the prepared hydrogel agents were stable without delamination and color change after centrifugation, and cold and heat resistance experiments (Figures 1E, F). The pore structure was observed by SEM (Figure 1G), all the hydrogels showed highly porous structure. The HA group had a macroporous structures, and the pore size of the HATA hydrogels gradually decreased with the increase of TA content. The quantitative results of pore size (Supplementary Figure S1) showed that the pore size of HATA hydrogels decreased from 85.83 ± 1.48 μm to 14.44 ± 1.56 μm. It was shown that the crosslink density of the hydrogels increased with increasing TA concentration, especially HATA 3 significantly increased the crosslinking density of the hydrogels. In addition, the connectivity between pores remained constant regardless of TA content, which indicated that the addition of TA maintained the hydrogel network. In addition, when treating acute eczema, the smaller the aperture of the hydrogel, the more effective the sustained-release treatment. This is because acute eczema is often accompanied by exudation, and a continuous and stable drug supply is needed to control skin inflammation.

The chemical changes of HATA hydrogels could be observed by FT-IR spectroscopy (Figure 1H). For pure TA, the broad bands between 3,500 and 3,000 cm^−1^ corresponded to the stretching vibrations of the phenol (-OH) group; the peak at 1714.95 cm^−1^ corresponded to the characteristic C=O stretching; the aromatic C=C stretching peaks appeared at 1,535.13, 1,447.55, and 1,031.05 cm^−1^; the aromatic moiety’s C-H bending vibration is shown by the peak at 758.64 cm^−1^. The TA-treated hydrogels showed overlapping TA signals compared to HA, demonstrating the successful integration of TA into the HA hydrogel network. Moreover, as TA content increased, the strength of TA’s intrinsic peak grew progressively, suggesting that TA and HA also formed a network. It is well known that intramolecular or intermolecular hydrogen bonding reduces the strength of the force constant of HA chemical bonding, which shifts the vibrational frequency to lower wavelengths. Stronger hydrogen bonds were generated as the TA content rose, resulting in chemical shifts from 3,443.51 to 3,432.36 cm^−1^ at lower wavelengths. This suggests that the HATA hydrogels form a physical cross-link through a hydrogen bond between the hydroxyl groups of HA and TA, and that these bonds increase with TA content; particularly, the HATA3 hydrogel shows a significant increase in hydrogen bonding.

The rheological properties of HA/TA hydrogels were analyzed by measuring the frequency scan and strain amplitude scan tests of HA/TA hydrogels with different TA contents (Shi et al., 2021; Zhou et al., 2023). In the swept frequency test (Figures 1I, J), the storage modulus (G′) value was larger than the corresponding loss modulus (G″) value in the frequency range of 0.1 ∼ 10 Hz, indicating that the HATA hydrogel has better stability. In addition, the G′ of the HATA hydrogel gradually increases with the increase of TA content. This is due to the increase in physical cross-linking by the addition of TA, which increases the mechanical strength of the hydrogel and induces strong interactions between the polymers. Figure 1K shows the results of the strain amplitude scanning tests. At the junction of the G′ and G″ curves, a 492.9% strain-time gel-sol transition occurred. From the above results, it can be obtained that the mechanical properties of HATA3 with the highest TA content are better than those of HATA2 and HATA1. Therefore, HATA3 with excellent mechanical properties was selected for the relevant experimental analysis in this paper.

### 3.2 Cytotoxicity and hemolysis assay

**FIGURE 2:**
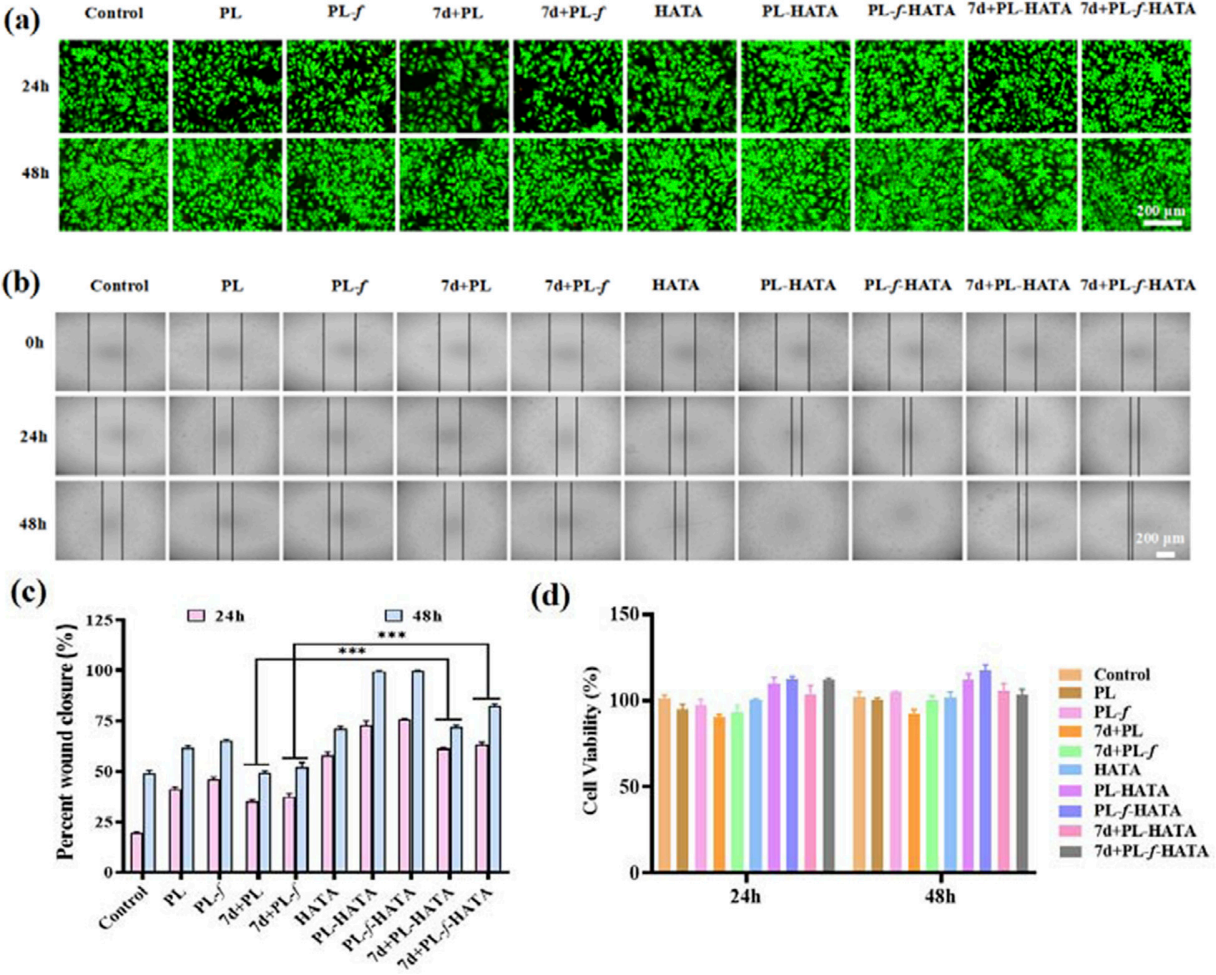
**(A)** Images of live or dead fluorescence labeling of HaCat cells co-incubated with various groups for 24 h and 48 h. **(B)** Cell migration images of different treatment groups in the scratch assay. **(C)** Quantitative evaluation of cell mobility of each group at 24 h and 48 h. **(D)** Relative activity of HaCat cells in different treatment groups. Scale bar = 200 μm (Analysis of differences between 7d+PL and 7d+PL-HATA groups, P*** < 0.001; Analysis of differences between 7d+PL-*f* and 7d+PL-*f*-HATA groups, P*** < 0.001).

MTT assay was used to validate the cytocompatibility of purslane hydrogels (Wang X. et al., 2023), the cytotoxicity of HaCaT cells treated with hydrogel for 24 and 48 h was determined. After 24 h (Figure 2D), all the hydrogels were non-toxic to the cells, and had promotion of cell growth, especially the PL-HATA and PL-*f*-HATA groups had a tendency to proliferate more obviously. After 48 h, the cell proliferation of PL-HATA and PL-*f*-HATA groups was obvious, and the survival rate reached 111.9% ± 0.49% and 117.4% ± 0.54%, which was mainly due to the flavonoids in purslane had the effect of promoting cell growth and proliferation. This was also demonstrated by co-staining of calcium flavin acetoxymethyl ester (AM) and propidium iodide (PI) (Figure 2A). Rapid cell migration is also necessary for cell wound healing, therefore, the effects of different treatments of Purslane hydrogels on HaCaT cell migration were further studied by cell scratch experiments. As can be seen by observing the cell scratch pictures (Figure 2B), after 24 h of cell migration, the scratch area of the hydrogels in each group was reduced to some extent. According to the quantitative data (Figure 2C), after 48 h, the healing rate of PL-HATA and PL-*f*-HATA scratches reached 99.5% ± 0.35%, 99.8% ± 0.21%, which showed excellent scratch healing activity and complete healing of cell wounds. The results indicated that the addition of PL-*f*, PL significantly improved the migration ability of HaCaT cells.

The degree of hemolysis is an important factor in assessing the biocompatibility of biomaterials that can come into direct contact with erythrocytes during application. The hemolytic toxicity of hydrogels was evaluated by measuring the hemolytic potential of red blood cells. The results showed that the hemolysis rate was less than 5% in all experimental groups (Supplementary Figure S2), which was in the safe range. The excellent hemocompatibility ensures the possibility of purslane hydrogels as wound-healing dressings. Therefore, these results suggest that purslane-loaded HATA hydrogels have excellent *in vivo* biocompatibility and are expected to realize applications in the field of skin dressings.

### 3.3 *In vitro* evaluation of antioxidant properties

**FIGURE 3:**
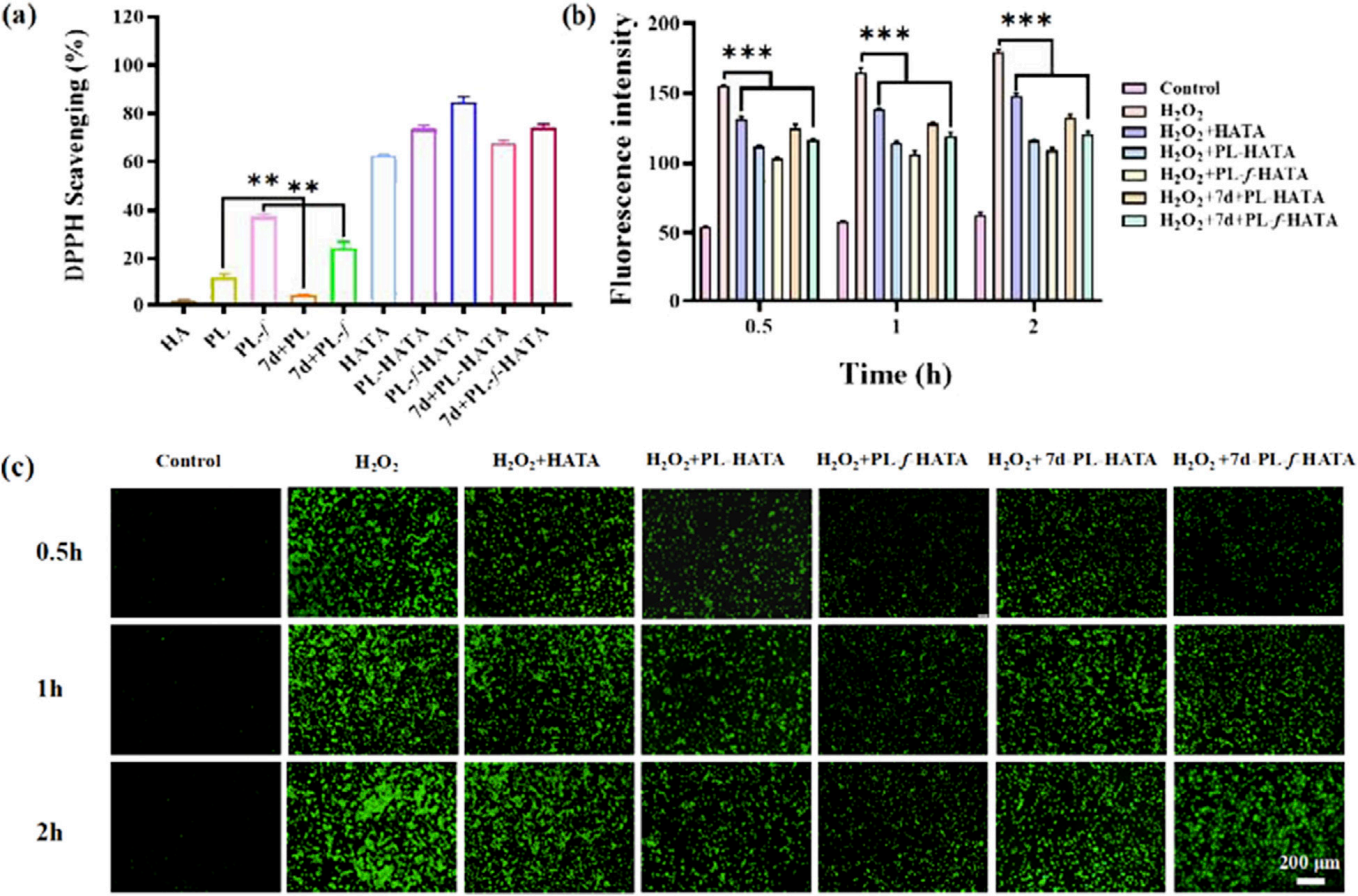
Antioxidant properties of hydrogels. **(A)** Experimental results of DPPH radical scavenging activity in different treatment groups. **(B)** Quantitative analysis of fluorescence intensity of induced ROS levels. **(C)** Fluorescence labeling of HaCaT cells with DCFH-DA probe and selection of representative images of ROS. Scale bar = 200 μm (data are expressed as mean ± SD (n = 3), *P < 0.05, **P < 0.01, ***P < 0.001).

Evaluation of antioxidant properties of purslane hydrogels by DPPH radical scavenging assay, when free radical scavenger was added to DPPH solution, the absorption disappeared or weakened when the lone pair electrons were paired, and the absorbance became smaller at 517 nm (Chang et al., 2023; Han et al., 2023). Monitoring the ultraviolet-visible spectral changes of DPPH solutions containing different samples for 60 min (Supplementary Figure S3), it can be seen that the absorbance of the pristine HA solution did not change much with time, whereas the absorbance of the HATA solution at 517 nm decreased rapidly. Especially, the introduction of purslane caused the gel absorbance to decrease even faster, indicating that purslane also contributes to free radical clearing ability. Then, the antioxidant activity of the hydrogels were evaluated by the change of absorbance at 517 nm (Figure 3A), with both HATA and purslane hydrogel groups showing high free radical scavenging abilities. Whereas HA itself had the least scavenging effect on the DPPH free radicals, indicating that scavenging effects were mainly from the TA fraction rather than the HA fraction. This is attributed to the fact that TA consists of pyrogallol and catechol moieties with a large number of hydroxyl groups, which gives TA a high free radical scavenging capacity through resonance stabilization of the off-domain electrons. In addition, purslane possesses free radical scavenging ability, attributed to its rich polyphenolic compounds, which exhibit strong antioxidant capacity to neutralize free radicals (Zhang et al., 2022; Wang Z. et al., 2023).

ROS play an important role in the pathogenesis of acute eczema (Park et al., 2020). Overexpression of ROS causes tissue damage, which leads to an increase in oxidative stress, resulting in oxidative damage to cell membranes, oxidative modification of DNA, and disruption of intracellular signaling pathways. It also results in disruption of the skin barrier, stimulates inflammatory responses, and hinders recovery from acute eczema. Purslane and TA both have antioxidant properties that can help neutralize free radicals and reduce oxidative damage (Zhang et al., 2023). To better confirm the ability of hydrogels to scavenge H_2_O_2_, the ROS content of the cells was determined in this work using the fluorescent probe DCFH-DA8. The higher the ROS content in the cells, the stronger the fluorescence intensity, so measuring the intensity of the fluorescence in each group allowed for the determination of the hydrogel’s antioxidant activity. As shown in Figure 3C, it was obvious that there was almost no fluorescence in the control group and ROS expression was extremely weak, while in the H_2_O_2_ group, fluorescence was significantly enhanced and ROS expression was strong, indicating that H_2_O_2_ induced intracellular ROS generation. In addition, the intensity of ROS expression in the HATA group was slightly decreased compared with that in the positive group, this is due to the elimination of ROS by TA, and the strong antioxidant properties of TA as a polyphenolic compound by itself. Due to the introduction of purslane, the expression of ROS in the 7d+PL-HATA and 7d+PL-*f*-HATA groups was further reduced, suggesting that purslane also has a certain ability to eliminate ROS. The fluorescence intensity in the PL-HATA and PL-*f*-HATA groups was the lowest and was maintained within a certain physiological range. Especially in the PL-f-HATA group, this was attributed to the fact that fresh purslane hydrogels contain high levels of purslane active ingredients and exhibit high antioxidant capacity, and meanwhile, the quantitative fluorescence data showed the same result (Figure 3B). Thus, it is evident that purslane hydrogels maintained the good survival of HaCat under oxidative stress by eliminating overexpressed ROS.

### 3.4 *In vitro* evaluation of antimicrobial properties

**FIGURE 4:**
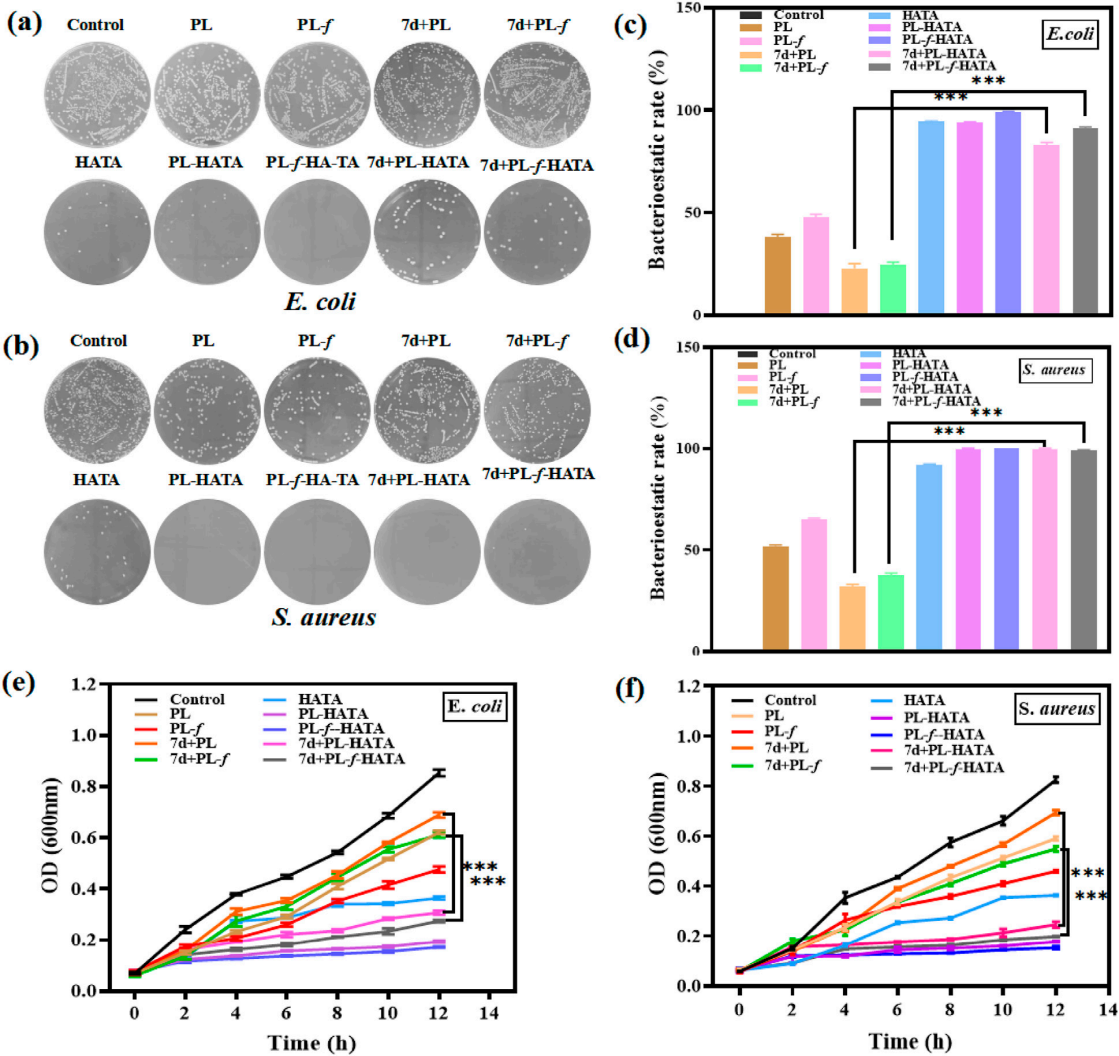
Antimicrobial evaluation of HATA hydrogels loaded with purslane. Pictures of plates demonstrating the suppression of *Staphylococcus aureus* **(B)** and *Escherichia coli* **(A)** activity following various sample treatments. Inhibition rates of *Escherichia coli* **(C)** and *Staphylococcus aureus* **(D)**. Growth curves of *Escherichia coli* **(E)** and *Staphylococcus aureus* **(F)** (Analysis of differences between 7d+PL and 7d+PL-HATA groups, P*** < 0.001; Analysis of differences between 7d+PL-*f* and 7d+PL-*f*-HATA groups, P*** < 0.001).

Bacterial infection is one of the most critical challenges in the recovery of acute eczema. Acute eczema is often inflamed and swollen or broken and is susceptible to microbial infections such as *E. coli* and *S. aureus*, which can cause itchiness and the appearance of papules, leading to exacerbation of the condition (Mei et al., 2022; Salle et al., 2024). In this study, the effect of hydrogel on bacterial activity was investigated on this issue. The results of plate counting experiments of *E. coli* and *S. aureus* showed (Figures 4A, B) that the antimicrobial effect of each experimental group was higher than that of the control group. The antimicrobial effect of the 4 groups of purslane hydrogels was significantly greater than that of the 4 groups of purslane liquid groups, among which the PL-HATA, PL-*f*-HATA groups had the highest antimicrobial activity, and the antimicrobial activity of the PL-*f*-HATA group was slightly superior to that of the PL-HATA. Quantitative data results (Figures 4C, D) also showed that the PL-*f*-HATA group could kill 99% ± 0.17% of *E. coli* and 100% ± 0.05% of *S. aureus*. It indicates that the gel dosage form retains the antibacterial activity of fresh purslane and has a significant inhibitory effect on *S. aureus* and *E. coli*, and the antibacterial effect of the gel dosage form can alleviate the symptoms of eczema.

In addition, bacterial growth was monitored by plotting the absorbance at 600 nm against the incubation time (Figures 4E, F). It can be seen that the OD values of purslane hydrogel groups were significantly lower than the purslane groups after 4 h of incubation, which indicates purslane hydrogels have a significant antibacterial effect. The FL-HATA group had the lowest OD value (E.*coli* 12 h OD value: 0.173 ± 0.002, S.*aureus* 12 h OD value: 0.152 ± 0.005), indicating that fresh purslane hydrogel can have a robust antibacterial effect.

**FIGURE 5:**
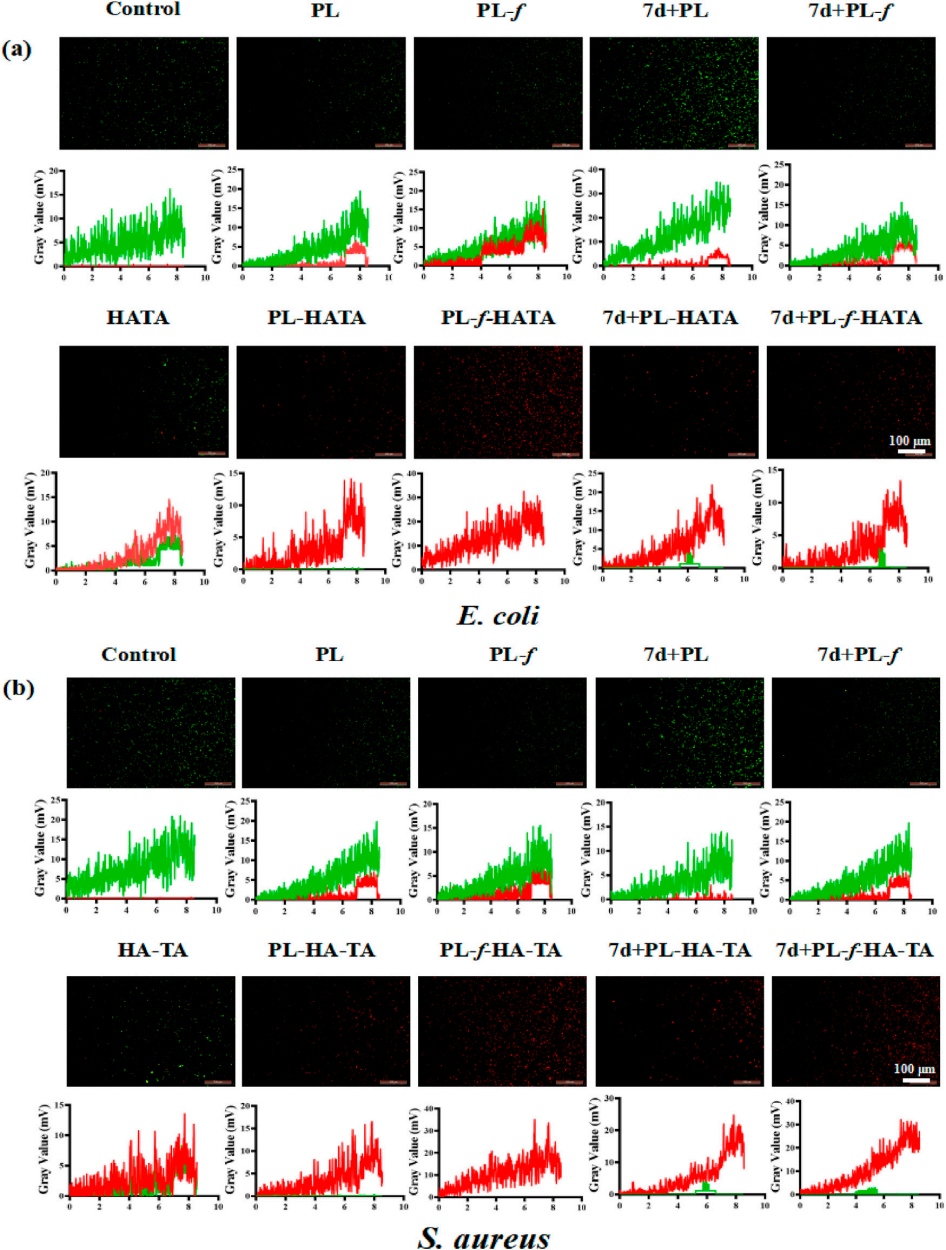
Representative live/dead fluorescence images of *Escherichia coli* **(A)** and *Staphylococcus aureus* **(B)** after co-incubation with PBS, PL, PL-*f*, 7d+PL, 7d+PL-*f*, HATA, PL-HATA, PL-*f*-HATA, 7d+PL-HATA, and 7d+PL-*f*-HATA for 12 h. Scale bar = 100 μm.

Next, the antimicrobial ability of purslane hydrogels was further explored by fluorescence staining experiments of live and dead bacteria. The fluorescent quantitation results (Figures 5A, B) showed that 7d+PL-HATA, 7d+PL-f-HATA, PL-HATA, PL-f-HATA groups exhibited red fluorescence and had a significant antibacterial effect. This agrees with the findings mentioned above for plate counting experiments.

### 3.5 Evaluation of skin damage in rats with acute eczema

**FIGURE 6:**
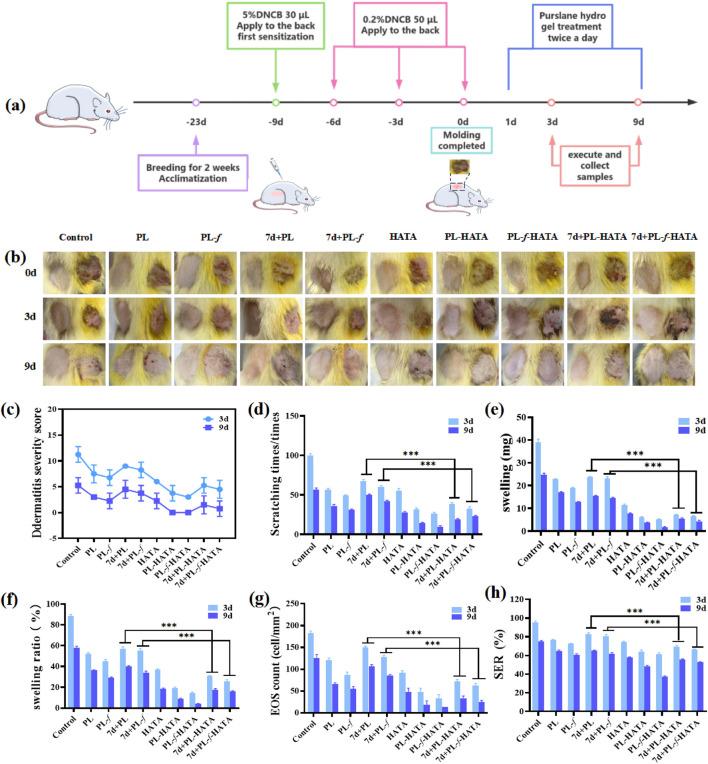
**(A)** Schematic diagram of the establishment of the rat model of acute eczema and the time points of drug treatment. **(B)** Photographs of acute eczema in rats at various time points after different groups of treatments. **(C)** Rat dermatitis scores. **(D)** Comparison of scratching behaviors of rats. **(E)** Comparisons of the skin swelling degree of the rats in each group on the third and ninth d of the treatment. **(F)** Comparison of inhibition rate of rats in each group on the 3d and 9d of the treatments. **(G)** Counts of eosinophils in the 3d and 9d of the treatments of the rats in each group. **(H)** Comparisons of skin roughness of the rats in each group at each time point (Analysis of differences between the 7d+PL and 7d+PL-HATA groups, P*** < 0.001; analysis of differences between the 7d+PL-*f* and 7d+PL-*f*-HATA groups, P*** < 0.001).

To assess the efficacy of using purslane hydrogels for the treatment of acute eczema, a rat model of acute eczema was prepared with DNCB induction for animal experiments. The success of the model was confirmed in combination with an increased percentage of eosinophils in the serum (Supplementary Figures S4, S5). The experimental groups were Control, PL, PL-*f*, 7d+PL, 7d+PL-*f*, HATA, PL-HATA, PL-*f*-HATA, 7d+PL-HATA and 7d+PL-*f*-HATA, and the control group was not treated. The experimental methods and treatments are shown in Figure 6A. The results of acute wet-type establishment in rats (Figure 6B) showed that at 0 d, redness, swelling, vesicular surface, and obvious plasma exudation appeared on the right back skin of each group after DNCB excitation, indicating that the modeling was successful. After 3 d of treatment, redness, swelling, and vesicles on the backs of each hydrogel group were reduced, and the highest treatment effect was observed in the PL-HATA, PL-*f*-HATA on the third day. What is more, the PL-HATA and PL-f-HATA groups demonstrated a significantly smoother eczema skin surface on day 9. This indicates that the combination of purslane with TA can serve to clear heat and remove toxins, astringe and reduce swelling, and decrease local exudation.

The dermatitis scores in rats in all experimental groups were reduced (Figure 6C), and the PL-f-HATA was 0 by day 9, which was significantly lower than those of other groups, which indicated that the fresh purslane hydrogels could to a great extent alleviate the symptoms of acute eczema. The results of scratching experiments (Figure 6D) showed that the number of rats scratching in the PL, PL-*f*, 7d+PL, and 7d+PL-*f* groups was significantly lower than that of the control group on the third and ninth days of treatment, indicating that purslane showed a better antipruritic effect. The HATA group also reduced the frequency of scratching to some degree. The PL-HATA and PL-f-HATA groups were able to reduce the symptoms of scratching considerably, with the PL-f-HATA group notably minimizing these symptoms. This indicates that the combination of PL-f and tannic acid could greatly reduce itching symptoms in rats. From the results of rat skin swelling (Figure 6E), it can be seen that compared with the model group, the degree of skin swelling of rats in each experimental group was significantly reduced. There was a significant difference between PL, PL-*f* and 7d+PL,7d+PL-*f*, while the degree of skin swelling in the hydrogel group was significantly reduced. Especially the PL-*f*-HATA group was almost swelling-free on the ninth day, which indicates that fresh purslane and tannic acid have astringent, anti-swelling and anti-inflammatory effects when combined.

The inhibition rate showed the same results (Figure 6F). The development of acute eczema increases the infiltration of EOS in the peripheral blood, and EOS is also involved in the pathogenesis of acute eczema. The relative count of EOS is also thought to correlate with the degree of itching in acute eczema, and it is also an independent parameter for assessing the severity of acute eczema (Inokuchi-Sakata et al., 2021; Čelakovská et al., 2023). As can be seen in Figure 6G, the number of EOS was significantly reduced in the PL-*f*-HATA group after treatment. Skin roughness is one of the common symptoms of acute eczema, which can be caused by weakening of sebaceous gland secretion function and prolonged scratching, which can impair the skin barrier function and is unfavorable to the recovery of eczema. Skin roughness can also be used as an indicator to evaluate the severity of acute eczema (Takada et al., 2022). As can be seen in Figure 6H, the skin roughness was reduced and maintained within the normal range by purslane hydrogels treatment, especially in the FL-HATA group, the lowest skin roughness was 37.12% ± 0.85% on day 9, which indicates that the treatment of FL-HATA can greatly alleviate the rough symptoms of eczema skin. The above results suggest that PL-*f*-HATA can reduce the symptoms of acute eczema and promote the recovery of skin damage.

### 3.6 Evaluation of the expression of inflammatory factors and immune factors in rats with acute eczema

The level of inflammatory factors in skin tissues can reflect the Th1/Th2 immune imbalance and inflammation. In the process of inflammation, anti-inflammatory factors and pro-inflammatory factors interact and antagonize each other in different links, and play a role in inhibiting inflammation. TNF-α is one of the pro-inflammatory factors, and the increase of TNF-α can cause skin damage, vasodilatation, increase of vascular wall permeability, chemotaxis of leukocytes, and pain-causing fever in the inflamed area, which will aggravate the inflammatory reaction. At the same time, the increase of TNF-α can also cause the increase of pro-inflammatory factors, such as IFN and IL-1, and further aggravate the inflammatory reaction of acute eczema skin lesions. Therefore, one of the pathways to exert anti-inflammatory effects in clinical practice is to downregulate the level of pro-inflammatory factor TNF-α. As a anti-inflammatory factor, IL-4 could not only regulate the differentiation and activation of T and B lymphocytes and promote the immune response characterized by Th2 cells but also play an anti-inflammatory role by inhibiting the production of TNF-α by mononuclear macrophages (Lee et al., 2020).

**FIGURE 7:**
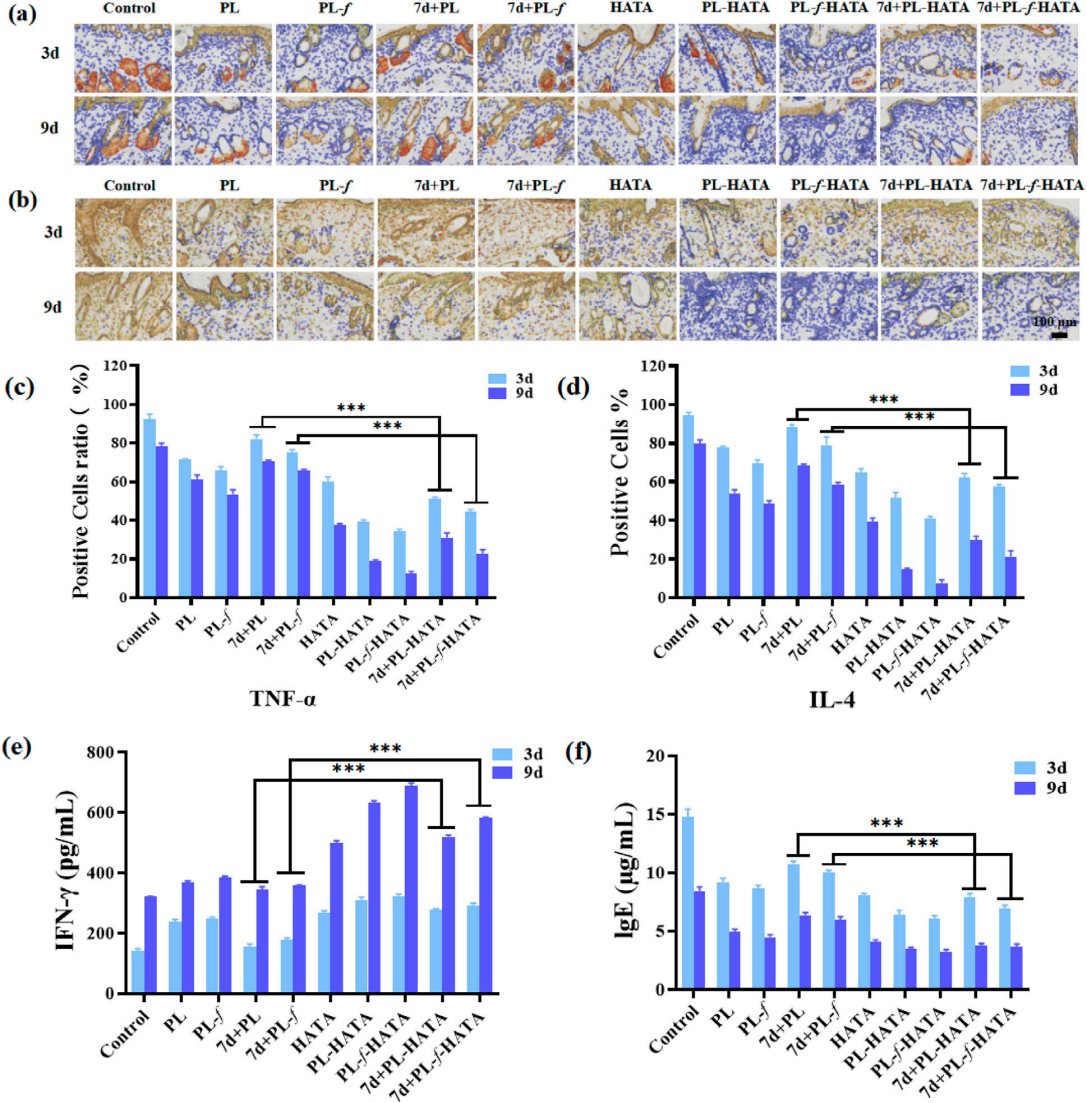
Representative immunohistochemical staining of TNF-α **(A)** and IL-4 **(B)** in skin tissues (scale bar = 200 μm). Comparison of the expression levels of TNF-α **(C)** and IL-4 **(D)** in the skin tissues of the rats in each group. Comparison of the expression levels of serum IFN-γ **(E)** and IgE **(F)** in the rats in each group (Analysis of differences between the 7d+PL and 7d+PL-HATA groups, P*** < 0.001; analysis of differences between the 7d+PL-*f* and 7d+PL-*f*-HATA groups, P*** < 0.001).

Immunohistochemical staining of TNF-α and IL-4 in rats for each group was observed under a microscope (Figures 7A, B). In Immunohistochemistry staining, positive substances refer to those that can specifically bind to particular antibodies and exhibit positive staining in tissue sections. The TNF-α and IL-4 positive substances were granular and filamentous, tan, brown, tan-yellow, yellow, and light yellow, and were located in the skin epidermal acanthocytes, skin appendage sweat gland cells, sebaceous cells, hair follicle cells, and cytoplasm of the endothelial inflammatory cells, and partially located in the endothelial mesenchymal tissue. On the third day of treatment, the TNF-α and IL-4 positive substances in the back skin tissues of rats in the control group with a wide and dense distribution area, TNF-α and IL-4 positive substances of purslane hydrogels groups were reduced. On the ninth day, the PL, PL-*f*, 7d+PL and 7d+PL-*f* groups stained yellow, light yellow, with a narrower and sparser distribution area, which indicates that purslane played an anti-inflammatory effect, mainly because the flavonoids and alkaloids in purslane can reduce the release of inflammatory factors, thus reducing the inflammation of the skin. In the PL, PL-*f* group, which is because the purslane medicine liquid has high active ingredients and a good anti-inflammatory effect. Moreover, the positive substances in the PL-f-HATA group were significantly reduced, indicating that purslane and TA are compatible and play an anti-inflammatory role. Consequently, the efficacy of the PL-f-HATA group in treating acute eczema was significant. The semi-quantitative TNF-α and IL-4 assays on the back skin of rats in each group also showed the same results (Figures 7C, D). At day 3, compared with the PL, PL-*f*, 7d+PL and 7d+PL-*f* groups, the four purslane hydrogels groups had a significant inhibitory effect on the skin tissues of rats with eczema, especially the PL-*f*-HATA group showed a significant decrease in the expression of TNF-α (12.62% ± 0.82%) and IL-4 (7.71% ± 1.34%) at day 9. The expression of TNF-α and IL-4 was significantly reduced in the PL-*f*-HATA group on day 9, indicating that the fresh product hydrogel had a significant anti-inflammatory effect.

Abnormal immunoregulation is a key trigger of eczema, especially because the dynamic imbalance of Th1/Th2 triggers cytokine secretion disorders, which play an important role in the occurrence and development of eczema. It was found that acute eczema patients have an imbalance in the ratio of Th2 cells to helper lymphocytes, and the level of IgE in the serum is abnormally elevated, resulting in immune abnormalities and triggering acute eczema. Th1 cells can secrete the immunomodulatory factor IFN-γ, which can inhibit the elevation of the level of IgE (Bing et al., 2017; Zhu Y. et al., 2023). IFN-γ is one of the most important factors that respond to the function of type 1 helper T cells, and the decrease in the level of expression of IFN-γ is closely related to the development of allergic diseases. IL-4 can also stimulate B lymphocytes to proliferate and synthesize IgE, and increase the expression level of IgE low-affinity receptor, which eventually leads to the increase of local skin vascular permeability, redness, swelling, itching, and other symptoms (Qiu et al., 2023). Therefore, in order to regulate the immune function, upregulation of IFN-γ and downregulation of IgE levels are required (Smolkova et al., 2017; Piipponen et al., 2020).

Compared with the control group, the expression level of IFN-γ in the serum of each experimental group did not show significant changes on the third day, while the expression of IgE was reduced to a certain extent (Figures 7E, F). On the ninth day of the treatment, the expression level of IFN-γ and IgE in the serum of each experimental group changed significantly. The expression level of IFN-γ in purslane hydrogels was higher than that in the control group, and the expression level of IgE was lower than that of the control group, especially in the PL-*f*-HATA group, which indicated that the combination of fresh purslane and TA could restore the immune imbalance of Th1/Th2 and regulate the immune response.

The above results show that the combination of fresh purslane and TA can reduce inflammation and regulate the immune function by inhibiting the expression of TNF-α, IL-4, and IgE, and increasing the expression of IFN-γ, which is effective in the treatment of acute eczema.

### 3.7 Histopathologic analysis of rat skin lesions

**FIGURE 8:**
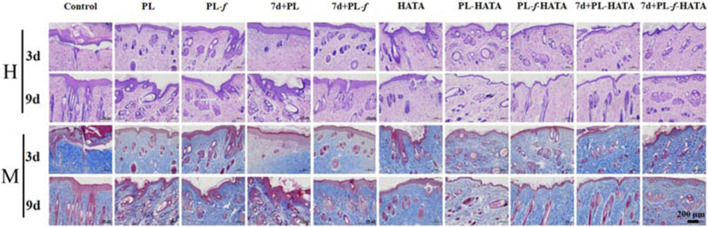
Histopathological changes of rat skin lesions in each group, H&E, and Masson staining analysis of histopathological sections of rat skin lesions on days 3 and 9 after different sample treatments (Scale bar = 200 μm).

Microscopic observation of skin pathological sections by H&E staining (Figure 8) showed that on the third day of treatment, the control group rats had increased thickness of the back skin tissue, epidermal lesions were detached, the granular layer was mildly hypertrophied, the sphenoid cells were edematous, and the inflammatory cell infiltration was obvious, which was consistent with the changes in the pathology of acute eczema. Purslane hydrogels group had mild edematous sphenoid cells and a small amount of inflammatory cell infiltration in the dermis, and the symptoms were significantly reduced compared with those of the control group. In purslane hydrogels, there was mild edema in the spinous layer cells, and a small amount of inflammatory cell infiltration in the dermis, which significantly reduced the symptoms compared with the control group. On the ninth day, the epidermal thickness of each experimental group was clearly reduced, while the epidermal and dermal structure of the purslane hydrogels groups was basically restored to intact, with normal thickness, the edema in the spinous layer cells was markedly improved, and the number of inflammatory cells in the dermis was obviously reduced. The PL-*f*-HATA group on the ninth day, the skin tissues basically recovered to normal, which indicated that purslane and TA had clear efficacy on acute eczema. In addition, Masson staining was used to analyze the production and distribution of collagen in dermal tissue. On the third day of treatment, the collagen fibers in the PL, PL-*f*, 7d+PL, and 7d+PL-*f* groups were unevenly spaced and arranged, while the collagen fibers in the 4 purslane hydrogels groups were increased in content and arranged in a regular manner, and the collagen synthesis was more frequent and regular in the freshly prepared PL-HATA and PL-*f*-HATA groups. On day 9, the collagen fibers in the PL, PL-*f*, 7d+PL, and 7d+PL-*f* groups were more disordered, while the collagen fibers in the PL-*f*-HATA group were increased, deeply stained, and more regularly arranged. The above results show that PL-*f*-HATA application can significantly reduce the inflammatory response of the skin tissues of rats with acute eczema and promote tissue repair, indicating that purslane fresh product gel has a clear efficacy on acute eczema.

### 3.8 Biocompatibility of purslane hydrogels

**FIGURE 9:**
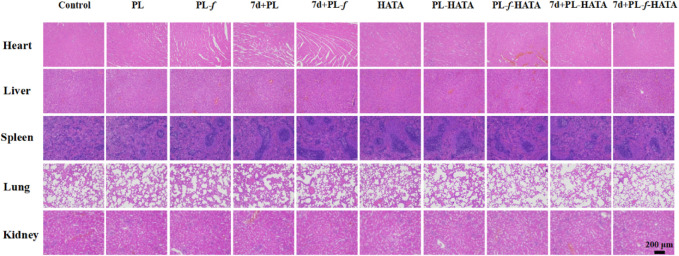
H&E analysis of major organs in SD rats after 9 days of treatment with different treatments (Scale bar = 200 μm).

Histocompatibility is an indicator of biosafety evaluation. For the treatment of acute eczema, the biosafety of the purslane hydrogels used is also an important indicator to examine whether it can be applied in the clinic. As shown in Figure 9, the five major organs of the SD rats treated with four groups of purslane hydrogels had no obvious tissue damage or lesions, indicating that the hydrogels has good biosafety.

## 4 Conclusion

In conclusion, the present study prepared a purslane gel formulation based on the compatibility theory between tannic acid and purslane. This formulation not only addresses the disadvantages of adverse reactions and drug dependence associated with Western drug treatments and the inconvenient operation of traditional Chinese medicine external therapies, but also overcomes the drawbacks of purslane’s easy oxidation and inconvenient use. Additionally, it enhances the storage stability and safety of purslane, and improves its therapeutic effect. Compared to purslane, the antimicrobial activity of the hydrogel is greatly enhanced by the antimicrobial properties of TA and retains excellent antimicrobial activity even after 7 days. Cellular experiments showed that purslane hydrogels had good biocompatibility, promoted cell proliferation and migration, and had a strong antioxidant capacity to maintain the balance of oxidative stress in the organism, the activity of purslane is well preserved during long-term storage. With the improvement of the dosage form and the compatibility between TA and purslane, the skin lesion score, scratching behavior, infiltration of EOS, swelling and inflammation level, immune response, and histopathological changes in rats were significantly improved, which also indicates that it has a therapeutic effect on rat eczema. This purslane application protocol solves the drawbacks of easy oxidation and inconvenience of use, improves its storage stability, and enhances its treatment effect. This is a key factor in further promoting the clinical application of purslane and provides a more valuable reference for future research and clinical application of purslane. It has important theoretical and practical significance to promoting the development of traditional Chinese medicine in China.

## Data Availability

The original contributions presented in the study are included in the article/Supplementary Material, further inquiries can be directed to the corresponding authors.

## Glossary

AM: calcium flavin acetoxymethyl ester
ANOVA: One-way analysis of variance
CFU: colony-forming units
DNCB: 2,4-dinitrochlorobenzene
DPPH: 1, 1-diphenyl-2-picrylhydrazyl
*E.coli*: E. *Escherichia coli*
EOS: eosinophils
G′: storage modulus
G″: loss modulus
H&E: hematoxylin and eosin staining
HA: hyaluronic acid
HATA: HATA hydrogel
IOD: optical density
LB agar: Luria-Bertani agar
LB broth: Luria-Bertani broth
MES: 2-(N-morpholino)ethanesulfonic acid
MT: Masson trichrome staining
OD: optical density
PI: propidium iodide
PL: traditional Chinese medicine purslane liquid
PL-*f*: fresh purslane medicinal liquid
7d+PL: traditional Chinese medicine purslane liquid placed for 7 days
7d+PL-*f*: fresh purslane medicinal liquid placed for 7 days
PL-*f-*HATA: PL-*f-*HATA hydrogel
PL-HATA: PL-HATA hydrogel
7d+PL-*f-*HATA: 7d+PL-*f-*HATA hydrogel
7d+PL-HATA: 7d+PL-HATA hydrogel
RBCs: Red blood cells
ROS: Reactive oxygen species
*S.aureus*: S. *Staphylococcus aureus*
SEM: scanning electron microscopy
TA: tannic acid
Th1/Th2: helper lymphocytes
