# Supplementary material for: A Chinese drug-compatibility-based approach to purslane hydrogels for acute eczema therapy
Source: Front Pharmacol. 2025 Feb 5;16:1504120. doi: 10.3389/fphar.2025.1504120 (PMC11841398; doi:10.3389/fphar.2025.1504120)
Supplement: Supplementary file 2 [file DataSheet3.docx]

**Supporting**

**
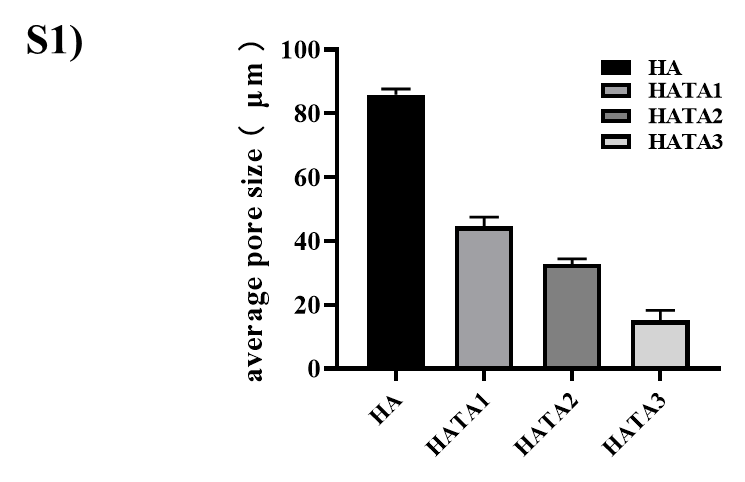
**

**Fig. S1** average pore size of HATA (molar ratios of 1:0, 1:2, 1:3 and 1:4) hydrogels;


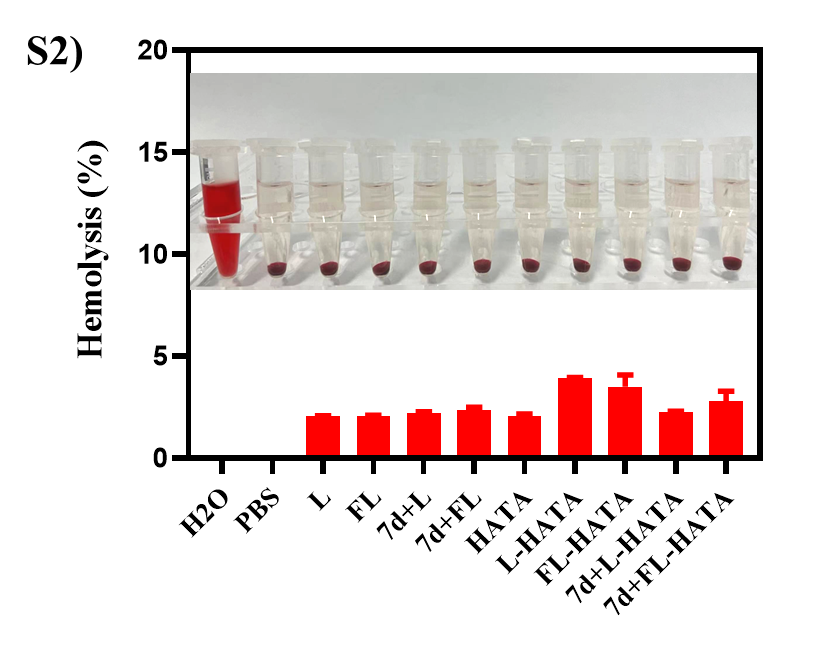


**Fig. S2** Hemolysis ratio of each experimental group;


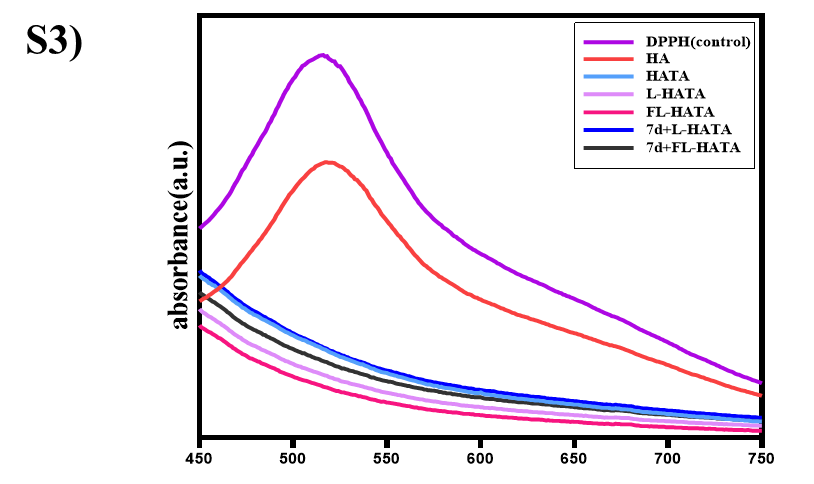


**Fig. S3** UV-visible spectra of DPPH solution after 60 min in the presence of HA and HATA samples;

**
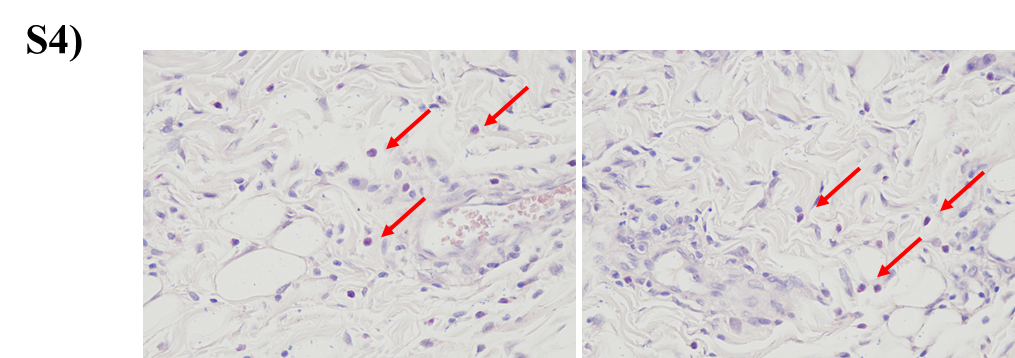
**

**Fig. S4** 0 d eosinophil pictures;


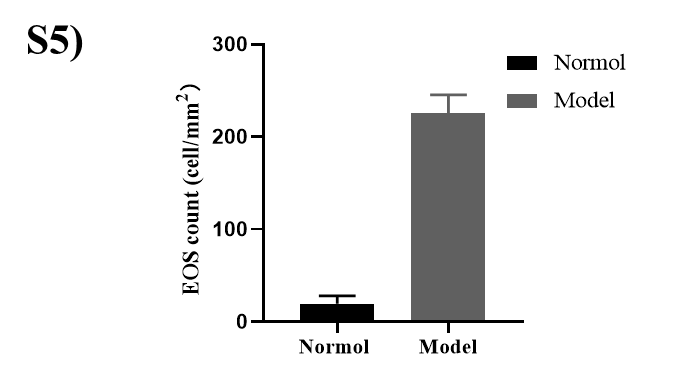


**Fig. S5** Counts of eosinophils in 0d rats.
